# Socio-demographic factors associated with incomplete vaccination or non-vaccination among children aged 12–23 months in Sierra Leone

**DOI:** 10.1186/s12889-025-24588-x

**Published:** 2025-10-09

**Authors:** Dove Djossaya, Thompson Igbu, Desmond Kangbai, Innocent Nuwagira, Andrew Kemoh, Michael Jones, Tom Sesay, Sallu Lansana, Samuel Adeyemi Williams, Gbessay Saffa, Ibrahim Franklyn Kamara, Adjidja Amani, Andre Arsene Bita Fouda, Blanche Philomène Melanga Anya

**Affiliations:** 1 Department of Demographic and Social Statistics, National Institute of Statistics, Yaounde, Cameroon; 2 World Health Organization Country Office, Communicable and Non-Communicable Disease Cluster, Freetown, Sierra Leone; 3 Directorate of Reproductive and Child Health Care, Ministry of Health, Freetown, Sierra Leone; 4 World Health Organization Country Office, Freetown, Sierra Leone; 5 World Health Organization Country Office, Universal Life Course Cluster, Freetown, Sierra Leone; 6 Department of Public Health, Faculty of Medicine and Biomedical Sciences, University of Yaounde I, Yaounde, Cameroon; 7 World Health Organization AFRO, Communicable and Non-Communicable Diseases, Vaccine Preventable Diseases, Brazzaville, Congo; 8 World Health Organization Country Office, N’Djamena, Chad; 9 College of Medicine and Allied Health Sciences, University of Sierra Leone, Freetown, Sierra Leone

**Keywords:** Incomplete Vaccination, Non-vaccination, Ascending Hierarchical Classification, Childhood Vaccination, Sierra Leone

## Abstract

**Background:**

Childhood vaccination coverage in Sierra Leone remains unsatisfactory despite multiple efforts made by health authorities to enhance collective immunity of the population, especially for children aged 12 to 23 months. This study aimed at identifying the factors associated with incomplete vaccination or non-vaccination among children aged 12 to 23 months in Sierra Leone in 2019.

**Methods:**

This was a cross-sectional study that used the 2019 Sierra Leone Demographic and Health Survey data. Descriptive statistics was performed to describe the sample, ascending hierarchical classification following a multiple correspondence analysis was employed to establish the profile of children with incomplete vaccination or non-vaccination status, and a binary logistic regression was used to identify the factors associated with incomplete vaccination or non-vaccination.

**Results:**

Of the 966 children aged 12 to 23 months involved in the study, 42.9% (95% CI[39.2; 46.6]) were incompletely vaccinated or unvaccinated, of which, 43.5% (95% CI[37.9; 49]) among male children and 42.4% (95% CI[37.3; 47.4]) among their female counterparts. The Northern (AOR 1.683, 95% CI[1.131; 2.503]) and the North Western (AOR 1.847, 95% CI[1.208; 2.825]) survey regions, delivery in a place other than a health facility (AOR 1.404, 95% CI[1.001; 2.042]), the mother’s age group 35 to 49 years (AOR 0.437, 95% CI[0.251; 0.762]) and the child’s birth order 7th or higher (AOR 2.640, 95% CI[1.452; 4.800]) turned out to be significant factors of incomplete vaccination or non-vaccination.

**Conclusion:**

Incomplete vaccination or non-vaccination among children was high in Sierra Leone in 2019. In order to increase childhood vaccine uptake, we recommend the development of initiatives geared towards optimizing facility-based service delivery, intensifying health education focusing more on vaccination, enhancing community engagement, addressing barriers to vaccine access, establishing and ensuring proper functioning of immunization defaulter tracking systems.

## Background

Vaccination is one of the most effective interventions in contemporary public health practices. Several cost–benefit analyses have consistently placed vaccination as one of the most cost-effective health initiatives [1]. It has substantially reduced the burden of vaccine-preventable diseases on children’s health. In 1980, the World Health Organization (WHO) officially declared the complete eradication of smallpox, the only human-based disease. This readily paved the way to foster the eradication of other vaccine-preventable diseases. In Africa, polio cases have been reduced by 99.9% globally since 1988 [2]. Approximately 23 million deaths were averted with the measles vaccine between 2010 and 2018 [3]. Every year, childhood vaccination prevents over 4 million deaths worldwide [4]. All of these successes have been achieved based on concerted efforts from countries, international organizations, non-governmental organizations and other vaccination-related partners.

Looking at the positive impact of vaccination not only on the population’s health, but also, on the economic and social aspects of countries, advocacy in favour of the development of vaccination-related activities have been on-going and intensified worldwide. The Global Vaccine Action Plan 2011–2020 (GVAP), endorsed by the 194 member states of the World Health Assembly in 2012, was a framework developed to help realize the vision of the decade of vaccines, which consisted in ensuring that all individuals and communities enjoy a life free from vaccine-preventable diseases. It was envisaged in this plan that countries by 2020 should have reached for their target populations, a national vaccination coverage of at least 90% and a district or equivalent administrative unit vaccination coverage of at least 80% [5]. With the emergence of the Covid-19 pandemic in late December 2019, several disruptions were observed across countries, notably the reduction of immunization services by 70% [6]. According to the New York Times, as of March 2021, more than 75% of Covid 19 vaccine doses were forwarded to wealthier countries, leading to an infringement of the vaccine equity policy. As such, WHO, the United Nations Children’s Fund (UNICEF), the Global Alliance for Vaccines and Immunization (GAVI) and other partners introduced in April 2021, the Immunization Agenda 2030 (IA2030), whose goals are designed to inspire action for implementation and support efforts to improve health security, universal health coverage, access and equity for immunization and innovation [7]. The IA2030 is built on successes of the GVAP and addresses its shortcomings to guide immunization efforts for the decade 2021–2030.

Despite the efforts of vaccination programmes and vaccination chain partners to improve the quality of vaccination services, notably through the introduction of new vaccines and the free access of some vaccines to specific age groups, some children still remain unvaccinated or have incomplete vaccination status. Globally, although there has been a slight recovery of vaccination coverage in 2022, about 20.5 million children are missing-out on life-saving vaccines, approximately 2.1 million more than in 2019 before the Covid-19 pandemic [8]. The Sub-Sahara African region has not witnessed any post-Covid-19 vaccination coverage recovery, with Diphtheria Pertusis and Tuberculosis 3 (DPT3) coverage stagnant at 74% and 64% respectively in the Eastern/Southern and Western African sub-regions in 2022 [8]. In Sierra Leone, according to findings of the Demographic and Health Survey (DHS), the proportion of children aged 12–23 months who received all basic vaccines recommended by the Expanded Program on Immunization (EPI) dropped between 2013 and 2019, varying from 68% [9] to 50.7% [10]. This situation may therefore tend to compromise herd immunity of children of this age group, thus increasing their risk of contracting diseases. The age bracket 12–23 months was considered here since all basic vaccines are generally administered to children before the age of 12 months and moreover, this will serve as a basis for comparison with findings from other settings. This study, which is the first of its kind, targeted children aged 12–23 months at the national level and had as major objective, the analysis of the socio-demographic factors associated with incomplete vaccination or non-vaccination. Specific objectives were: determining prevalence of incomplete vaccination or non-vaccination, identifying factors of incomplete vaccination or non-vaccination and establishing a profile of children unvaccinated or with incomplete vaccination status.

## Methods

### Study design

This was a cross-sectional study that used the 2019 Sierra Leone Demographic and Health Survey (SLDHS) data. The 2019 SLDHS implemented a two-stage stratified probability cluster sampling methodology. Stratification variable was the area of residence, comprised of the urban and rural areas. In the first stage, Enumeration Areas (EAs) were selected using probability proportional to size. In the second stage, households were selected using systematic random sampling.

### Study setting

The scope of the study was Sierra Leone and analysis was conducted for each of the five administrative regions of the country, namely, the Northern, Eastern, Western, Southern and the North Western. Sierra Leone is a country located in West Africa. It is bordered in the West by the Atlantic Ocean, in the North and North East by Guinea Conakry, and in the South and South East by Liberia. It covers a total surface area of 71,740 square kilometres and had a projected population of 8,969,980 inhabitants in 2024 [11].

### Participants

All live children aged 12–23 months born in the 3 years preceding the survey were included in this study. A child was considered with incomplete vaccination status if the later failed to receive at least one of the following vaccines: Bacillus Calmette-Guérin (BCG), three doses each of polio and pentavalent, and one dose of measles-containing vaccine. Moreover, a child was considered unvaccinated if he or she has not received any of the above-mentioned vaccines.

### Variables

The independent variables used in this study were: mother’s education, mother’s age group, mother’s marital status, mother’s access to media, mother’s antenatal care, place of delivery, child’s birth order, child’s sex, household wealth index, survey region and area of residence. The dependent variable was the non-vaccination or incomplete vaccination status of the child.

### Data source

This study used secondary data from the 2019 Sierra Leone Demographic and Health Survey, a nationwide household-based survey.

### Study size

During the 2019 SLDHS, 578 EAs were covered, among which, 13, 399 households interviewed during data collection. In each household, all women aged 15–49 years were interviewed and in 1 household out of 2, all men aged 15–59 years were interviewed. Questions on children’s health and more specifically, those related to vaccination were asked to all mothers who had a live birth in the 3 years preceding the survey, considered as the reference period of the study. Altogether, 966 children aged 12–23 months were involved in the study.

### Statistical methods

Data cleaning and tabulation was done using SPPS version 22 to facilitate output exportation and presentation, while data visualisation, logistic regression, ascending hierarchical classification following multiple correspondence analysis were implemented using R version 4.0.0 as the latter uses an extensive collection of packages that enhance quality of analyses. Descriptive statistics was performed to describe the sample and to estimate the prevalence of incomplete vaccination or non-vaccination, backward stepwise binary logistic regression was carried out to identify the explanatory factors of incomplete vaccination or non-vaccination, ascending hierarchical classification following a multiple correspondence analysis was employed to establish the profile of children with incomplete vaccination or non-vaccination status. Validation of the logistic regression model was made possible using the likelihood ratio test to assess the overall model significance, the McFadden pseudo R^2^ for goodness of fit and the Hosmer and Lemeshow test for model specification. Multicollinearity was handled using the Generalized Variance Inflating Factor (GVIF). Covariates with corresponding GVIF values greater than 5 were considered highly correlated and thus non informative in the presence of other covariates. Covariates with p-values less than 5% were considered statistically significant. Missing data was handled using multiple imputation method via mice, lattice and VIM packages in R. Weighted data analysis was conducted to ensure generalization and representativeness of results.

## Results

### Descriptive data

Out of 966 children aged 12–23 months included in this study, 50.1% (*n* = 484) were females and 49.9% (*n* = 482) were males. Their average and median ages were 16.7 months and 16 months respectively. The spatial distribution of children throughout the country showed that most of them were based in the rural settings (72.5%, *n* = 700). The Southern region had the highest number of children with 26.1% (*n* = 250) of cases, while, the Western region had the lowest, with only 11.3% (*n* = 109). Moreover, a greater proportion of children were delivered within a hospital environment, with virtually 9 children out of 10 delivered in a health facility. The study sample also had numerous older births and fewer recent ones. Indeed, more than half of the children occupied the 1 st to the 3rd birth positions (60.9%, *n* = 588), while only about 8% occupied the 7th birth position or higher (Table 1).

**Table 1:** Distribution (in %) of children aged 12–23 months by selected sociodemographic characteristics

Variable	Number (n)	Percent (%)
**Survey region**		
Eastern	190	19.7
Northern	233	24.1
North Western	182	18.8
Southern	252	26.1
Western	109	11.3
Total	966	100.0
**Area of residence**		
Urban	266	27.5
Rural	700	72.5
Total	966	100.0
**Child’s sex**		
Male	482	49.9
Female	484	50.1
Total	966	100.0
**Place of delivery**		
In a health facility	823	85.2
Not in a health facility	143	14.8
Total	966	100.0
**Birth order**		
1 st −3rd order	588	60.9
4th −6th order	302	31.3
7th order or +	76	7.9
Total	966	100.0
**Mean age: 16.7 months**		
**Median age: 16 months**		

### Outcome data

The proportion of children aged 12–23 months unvaccinated or with incomplete vaccination status was estimated at 42.9% (95% CI [39.2; 46.6]), with 43.5% for male children and 43.4% for their female counter parts. This proportion was slightly higher in the rural area (44.3%, 95% CI [34.1; 43.3]) than in the urban area (39.6%, 95% CI [48.3; 58.8]). The analysis of this proportion by survey region clearly showed that children unvaccinated or with incomplete vaccination status were mostly located in the Northern parts of the country, with the North Western region (52.5%, 95% CI [44; 60.9]) having the highest proportion, followed by the Northern region (48.8%, 95% CI [40; 57.6]). The lowest proportion was however observed in the Southern region (32.4%, 95% CI [25.4; 39.3]) (Table 2).

**Table 2:** Proportion of children aged 12–23 months with incomplete vaccination status or unvaccinated by region, area of residence and child’s sex

	**Estimate**	**Standard Error**	**95% Confidence Interval**		**Design Effect**	**Number of children (weighted)**
			**Lower bound**	**Upper bound**		
**Survey region**						
Eastern	38.7	4.076	30.7	46.7	1.423	199
Northern	48.8	4.459	40.0	57.6	1.629	200
North Western	52.5	4.289	44.0	60.9	1.385	183
Southern	32.4	3.532	25.4	39.3	1.219	209
Western	43.5	5.143	33.4	53.6	1.673	152
**Area of residence**						
Urban	39.6	3.394	33.0	46.3	1.400	284
Rural	44.3	2.291	39.8	48.8	1.434	659
**Child’s sex**						
Male	43.5	2.824	37.9	49.0	1.531	482
Female	42.4	2.583	37.3	47.4	1.348	484
Total	42.9	1.898	39.2	46.6	1.419	943

**Fig. 1:**
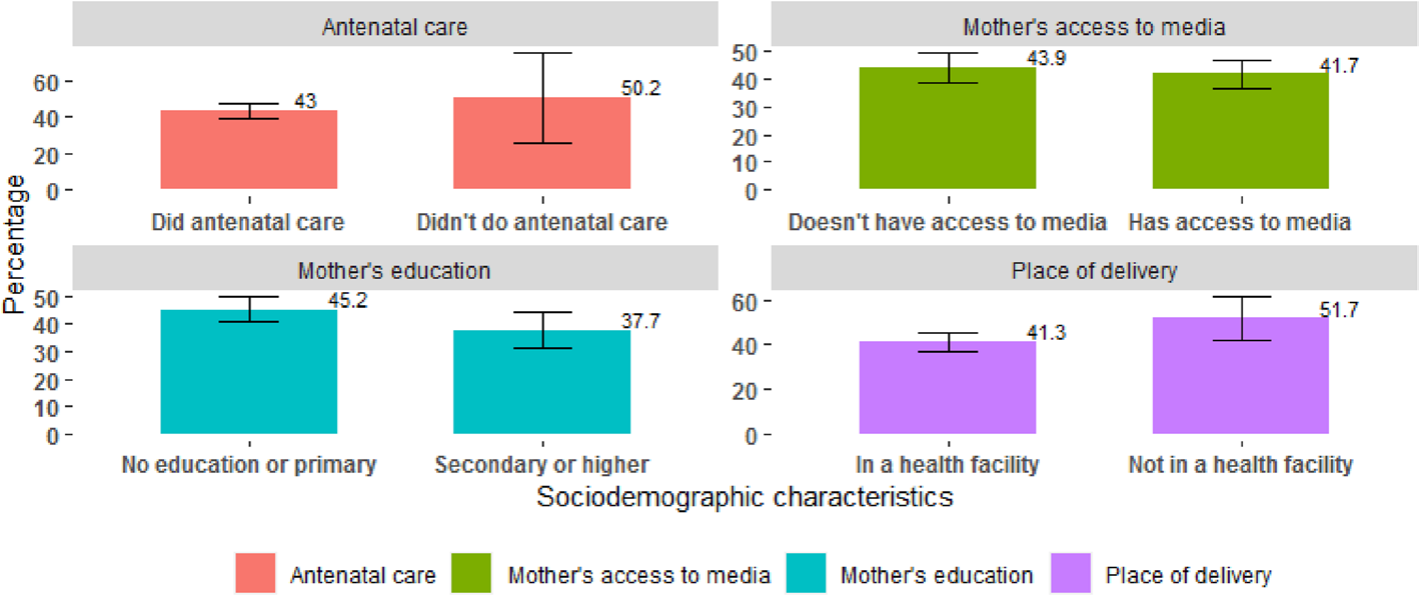
Proportion of children aged 12–23 months with incomplete vaccination status or unvaccinated by selected sociodemographic characteristics

Incomplete vaccination or non-vaccination of children decreased with increase in education level of mothers. The proportion of children aged 12–23 months with incomplete vaccination status or unvaccinated stood at 37.7% (95% CI [31.4; 44.1]) among children whose mothers had a secondary education level or higher, as compared to 45.2% (95% CI [40.9; 49.5]) among those whose mothers were uneducated or basically had the primary education level. Incomplete vaccination or non-vaccination also varied with the place of delivery. Indeed, the proportion of children with incomplete vaccination status or unvaccinated was lower among children who were delivered in a hospital setting (41.3% (95% CI [37.3; 45.4])) than among those who were delivered in a non-hospital setting (51.7% (95% CI [42.3; 61])). This proportion decreased from 50.2% (95% CI [26.3; 74.1]) among children whose mothers did not attend antenatal care during pregnancy to 43% (95% CI [39.1; 46.8]) among those whose mothers attended antenatal care (Fig. 1).

Results from the ascending hierarchical classification [12] following multiple correspondence analysis [13] enabled the establishment of three main groups of children with incomplete vaccination status or who were unvaccinated as described below.

### Main results

#### Group 1

This group is characterized by children with relatively high incomplete vaccination or non-vaccination status. These children were born in a hospital setting, mostly reside in the rural areas of the Northern and North Western regions of the country and come from poor households. Their mothers have the primary education level or are uneducated and are generally young with an age varying from 15–20 years.

#### Group 2

This is the intermediate group. Children in this group have moderate incomplete vaccination or non-vaccination levels. They mostly reside in the urban areas of the Western and Eastern regions of the country and come from non-poor households. Their mothers have the primary education level or are uneducated and are generally adults of age 21–34 years, who have undergone antenatal care visits during pregnancy.

#### Group 3

**Fig. 2:**
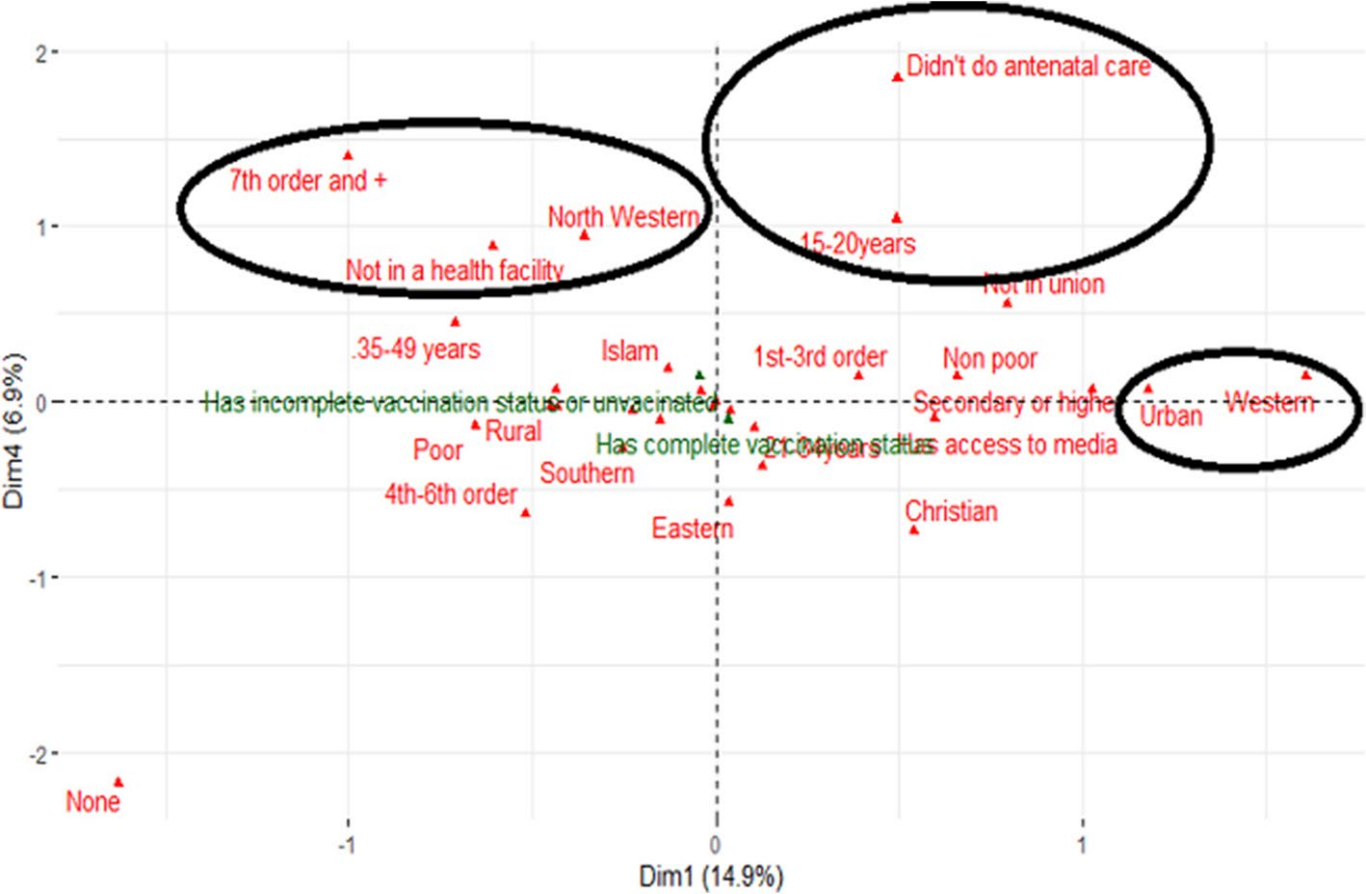
Multiple correspondence analysis dimensional plot

Considered as the class with relatively low incomplete vaccination or non-vaccination, this group brings together children who mostly reside in the Southern part of the country and whose mothers have attended secondary or higher education and who are quite mature, with ages varying from 35–49 years (Fig. 2).

Findings from the multivariate analysis [14] showed that the logistic regression model performed was globally significant, with a case classification power of about 63%. The survey region, the child’s birth order, the child’s place of delivery and the mother’s age group were significant factors of incomplete vaccination or non-vaccination of children. Children born to mothers aged 35–49 years are about 0.5 times less likely of being unvaccinated or having an incomplete vaccination status than those born to mothers aged 15–20 years (Table 3).

**Table 3:** Binary logistic regression model output

Variable	P value	Adjusted odds ratio	95% C.I. for the adjusted odds ratio	
			**Lower bound**	**Upper bound**
**Place of delivery**				
In a health facility (Reference)				
Not in a health facility	0.076**	1.404	1.001	2.042
**Birth Order**				
1st-3rd order (Reference)				
4th-6th order	0.155	1.269	0.914	1.762
7th + order	0.001*	2.640	1.452	4.800
**Mother's age group**				
15-20Yrs (Reference)				
21-34Yrs	0.123	0.729	0.487	1.090
35-49Yrs	0.003*	0.437	0.251	0.762
**Survey region**				
Eastern (Reference)				
Northern	0.010*	1.683	1.131	2.503
North Western	0.005*	1.847	1.208	2.825
Western	0.113	0.723	0.483	1.080
Southern	0.247	1.335	0.818	2.179
**Mother's marital status**				
In union (Reference)				
Not in union	0.218	1.275	0.866	1.875
**Mother's education**				
No education or primary (Reference)				
Secondary or higher	0.086	0.749	0.538	1.041
**Wealth index**				
Poor (Reference)				
Non-poor	0.936	1.012	0.753	1.360
**Child’s sex**				
Male (Reference)				
Female	0.854	0.975	0.749	1.270

^*^P value < = 0.05

^**^P value < = 0.1

Additionally, it was found that the likelihood of incomplete vaccination or non-vaccination increases by 40% among children who were not born in a health facility, compared to those who were born in a health facility. Children who occupied the 7th birth position or higher are about three times more likely of being unvaccinated or incompletely vaccinated than those who occupied the 1 st to the 3rd birth positions. The odds of being incompletely vaccinated or unvaccinated increases by 85% and 68% respectively among children residing in the North Western and the Northern regions compared to those residing in the Eastern region [15].

## Discussion

Among all the children aged 12–23 months involved in the study, close to half (42.9%) were unvaccinated or had their vaccination incomplete, with a higher proportion observed in the rural area (44.3%), compared to the urban area (39.36%). Exploratory analysis conducted helped to classify children with incomplete vaccination or non-vaccination status in to 3 groups, namely, children with high, medium or low incomplete vaccination or non-vaccination levels. This classification illustrated that children with high incomplete vaccination or non-vaccination level mostly resided in the Northern and the North Western regions of the country. In the same vein, result from econometric analysis showed that there is a high propensity for children residing in the Northern and the North Western regions of being unvaccinated or incompletely vaccinated. This finding is in agreement with a study conducted in Pakistan in 2023, that showed the association between the region of residence and incomplete or non-vaccination of children. According to this study, children living in Fata and Balochistan regions were identified of being at highest risk of incomplete vaccination, compared to those living in Punjab [16]. Based on this finding, it is advisable that more attention should be given by vaccination officials to these regions during national routine and supplementary immunization activities, in order to bridge the gap of vaccination imbalance between regions. Furthermore, actions to raise awareness on the importance of childhood vaccination among mothers, and more specifically those originating from these two regions, should be undertaken in order to enhance vaccination service performances.

It was also observed that giving birth in a place other than a health facility increases the risk for a child of being unvaccinated or incompletely vaccinated. This result is in line with the findings obtained in 2015 from a study conducted in six countries of West Africa using data from the DHS. In that study, it was demonstrated that home birth was a risk factor for incomplete vaccination among children aged 12 to 59 months [17]. Additionally, this finding aligns with those of the study conducted in four other countries in West Africa, where it was illustrated that children born in health facilities have a lower probability of incomplete vaccination compared to those born at home [18]. One possible reason underlying this situation is that, once a new-born is delivered at home or in an environment outside a health establishment, there is no contact between the mother and the health professional, and this could obviously influence the mother's knowledge about post-natal care, including childhood vaccination schedule. Furthermore, it is possible that these mothers might have not been attending antenatal care and this can also compromise their knowledge about childhood vaccination.

The conclusions of the study also stipulated that mother's age group significantly explains incomplete vaccination or non-vaccination of children. Being a mature mother (35–49 years) reduces the risk for a child to be incompletely vaccinated or unvaccinated. An identical result was obtained in a study conducted in Ghana in 2016, where it was shown that children born to older mothers (40–49 years) were more likely to be vaccinated than those born to relatively younger mothers (under 20 years) [19].

This is also consistent with the study of Olorunfemi et al. conducted in 2025 in West Africa, where it was shown that children born to older mothers had lower odds of having incomplete vaccination than those born to younger ones [18]. This conclusion raises the issue of early childbearing and its consequences not only at the family level, but also, at the societal level in general. Teenage mothers, due to their youthfulness and lack of experience, can face difficulties in performing their motherhood duties, including the handling of childhood vaccination schedule. In Sierra Leone, early childbearing is still a concern as this was illustrated with the 2019 findings of the DHS, where 21% of teenagers (15–19 years) had already given birth or were pregnant with their first child at the time of data collection [10]. Ignorance, immaturity, lack of adequate information on vaccination and to some extent, social stigmatization of young mothers could be potential factors why their kids are more exposed to incomplete vaccination or non-vaccination. Measures such as motivating women in education as an alternative to early marriage and also promoting communication, education and public awareness programmes regarding vaccination should be encouraged.

The child’s birth order was also another significant factor of incomplete vaccine or non-vaccination. Children of the 7th birth order or higher were more likely to have an in complete vaccination status or to be unvaccinated. This result is supported by a previous study conducted in Ethiopia, where it was proven that children with later birth orders had more odds to be incompletely vaccinated [20]. Zenbaba et al. [21] equally arrived to the same conclusions. This situation could be explained by the fact that as the number of children in the family increases, the resources, including time and parental attention, are shared among the children. Consequently, this may result in incomplete vaccination status or absence of vaccination of later born children in the family.

### Strengths and limitations

This study presents a number of strengths. First and foremost, it used a two-stage probability cluster sampling methodology, which is a new standardized methodology designed by WHO for vaccination coverage survey estimates [22] [23]. Secondly, the findings of the study have a broader scope, with estimates targeting both the national and the sub national levels. Thirdly, the DHS data source used for this study is a standardized source that can help to compare findings over time and across different countries in Africa and the globe as a whole.

Despite the results obtained in this study, a number of limitations were outlined. Firstly, in the absence of vaccination records, information on child immunization was collected on the basis of the mother's declaration, which is subject to recall bias when estimating parameters. Vaccination data collected through vaccination records is more reliable and makes it possible to obtain accurate estimates, reflecting the real situation on the ground. Ensuring massive distribution of vaccination booklets or cards and organizing frequent awareness-raising campaigns in communities to explain the importance of children’s vaccination will help to improve vaccination card or booklet retention. Secondly, the sample size for children aged 12–23 months used for this study was not large enough to compute estimates for each of the 16 districts of the country. Estimates were rather computed for the major five regions of the country.

## Conclusion

Incomplete vaccination or non-vaccination of children aged 12-23 months was high in Sierra Leone in 2019. Indeed, the proportion of children aged 12–23 months unvaccinated or with an incomplete vaccination status stood at 42.9% (95% CI [39.2; 46.6]), with some disparities observed at the regional level. Several factors were associated with incomplete vaccination or non-vaccination. These include the survey region, child delivery outside a health facility, mother’s age group (35 to 49 years) and the child’s birth order 7th or higher. Taking the aforementioned variables into account in the elaboration of health policies and strategies and more specifically, those pertaining to childhood vaccination, would contribute to scale-down the burden of incomplete vaccination or non-vaccination of children, thus leading to increase in vaccination coverage. As facility delivery is high globally, the following measures could be observed in view of scaling-up childhood vaccine uptake: optimizing facility-based service delivery, intensifying health education focusing more on vaccination, enhancing community engagement, addressing barriers to vaccine access, establishing and ensuring proper functioning of immunization defaulter tracking systems. Having observed that most of the predictors are linked to mother and child individual characteristics, it would be useful to undertake a study that has a broader spectrum, covering not only socio-demographic components, but also, structural factors relative to the provision of health services. In this regards, a facility-based study, coupled with a qualitative component could also be envisaged in order to provide complementary findings regarding this topic.

## Data Availability

The data used for this study was obtained from the DHS Program website. The DHS Program gave approval to access the data and to use it for the sole purpose of the study. (https://dhsprogram.com/data/dataset/Sierra-Leone_Standard-DHS_2019.cfm?flag = 1).

## Abbreviations

AOR: Adjusted odds ratio
BCG: Bacillus calmette-guérin
CI: Confidence interval
DHS: Demographic and health survey
DPT: Diphtheria pertusis tuberculosis
EA: Enumeration area
EPI: Expanded program on immunization
GAVI: Global alliance for vaccines and immunization
GVAP: Global vaccine action plan
GVIF: Generalized variance inflating factor
SLDHS: Sierra leone demographic and health survey
UNICEF: United nations children’s fund
WHO: World health organization
